# A modified generalized Fisher method for combining probabilities from dependent tests

**DOI:** 10.3389/fgene.2014.00032

**Published:** 2014-02-20

**Authors:** Hongying Dai, J. Steven Leeder, Yuehua Cui

**Affiliations:** ^1^Department of Pediatrics, Research Development and Clinical Investigation, Children's Mercy HospitalKansas City, MO, USA; ^2^Department of Pediatrics, University of Missouri-Kansas CityKansas City, MO, USA; ^3^Department of Informatic Medicine and Personalized Health, University of Missouri-Kansas CityKansas City, MO, USA; ^4^Department of Pediatrics, Clinical Pharmacology and Therapeutic Innovation, Children's Mercy HospitalKansas City, MO, USA; ^5^Department of Statistics and Probability, Michigan State UniversityEast Lansing, MI, USA

**Keywords:** generalized Fisher method (Lancaster procedure), weight function, correlated *p*-values, multiple hypothesis testing, high dimensional genetic data

## Abstract

Rapid developments in molecular technology have yielded a large amount of high throughput genetic data to understand the mechanism for complex traits. The increase of genetic variants requires hundreds and thousands of statistical tests to be performed simultaneously in analysis, which poses a challenge to control the overall Type I error rate. Combining *p*-values from multiple hypothesis testing has shown promise for aggregating effects in high-dimensional genetic data analysis. Several *p*-value combining methods have been developed and applied to genetic data; see Dai et al. (2012b) for a comprehensive review. However, there is a lack of investigations conducted for dependent genetic data, especially for weighted *p*-value combining methods. Single nucleotide polymorphisms (SNPs) are often correlated due to linkage disequilibrium (LD). Other genetic data, including variants from next generation sequencing, gene expression levels measured by microarray, protein and DNA methylation data, etc. also contain complex correlation structures. Ignoring correlation structures among genetic variants may lead to severe inflation of Type I error rates for omnibus testing of *p*-values. In this work, we propose modifications to the Lancaster procedure by taking the correlation structure among *p*-values into account. The weight function in the Lancaster procedure allows meaningful biological information to be incorporated into the statistical analysis, which can increase the power of the statistical testing and/or remove the bias in the process. Extensive empirical assessments demonstrate that the modified Lancaster procedure largely reduces the Type I error rates due to correlation among *p*-values, and retains considerable power to detect signals among *p*-values. We applied our method to reassess published renal transplant data, and identified a novel association between B cell pathways and allograft tolerance.

## Introduction

Rapid developments in molecular technology have created high throughput data in search of genetic variants associated with complex traits. As the cost of experiments goes down, the amount of data that can be generated, and the resulting complexity of statistical analysis required to interpret the data goes up. The increase of genetic variants requires more statistical testing to be performed simultaneously, which poses a challenge to control the genome wide Type I error rate. False discovery rate (FDR) and its extended methods have been proposed to adjust *p*-values in multiple tests in order to control the genome wide Type I error (Benjamini and Hochberg, 1995; Cheng and Pounds, 2007). However, in large-scale hypothesis testing, these methods often require very a large sample size to maintain power of detecting risk factors.

The global test (also named omnibus test) of *p*-values can combine evidence and turn dimensionality from a curse into rich information. From a systems biology perspective, genes, cells, tissues, and organs function as a system through metabolic networks and cell signaling networks. In non-Mendelian inheritance patterns, such as complex disorders, a subset of genetic variants may jointly confer moderate effects in mediating molecular activities. As a result, signals may not be significant in single marker-single trait analysis, but many such values from related genes might provide valuable information on gene function and regulation. For instance, in pathway analysis (Khatri et al., 2012) and gene set enrichment analysis (Subramanian et al., 2005), multiple genes that work together to serve a particular biological function are often analyzed jointly as a gene set. Several pathway repositories, such as the Kyoto Encyclopedia of Genes and Genomes (KEGG) (Kanehisa et al., 2004), PANTHER classification system for protein sequence data (Nikolsky and Bryant, 2009), and Reactome pathways in humans (Matthews et al., 2009) have been established, and are continually being updated. For non-Mendelian diseases and complex traits, identification of isolated genetic variants is insufficient to summarize the complex association with disease. The “most-significant SNPs/genes” approach often detects variants with small effect sizes and odds ratios ranging between 1.3 and 2 (Wacholder et al., 2004). Therefore, integrating information from pathways, gene sets, and networks will provide useful information in understanding the gene regulation mechanism. Furthermore, filtration techniques can be integrated with global testing of *p*-values to remove sets of genetic variants that are not related to traits, and thereby reduce the dimensionality of the data (Dai and Charnigo, 2008; Dai et al., 2012a).

The global test of *p*-values evaluates the pattern (distribution) of *p*-values instead of selecting *p*-values less than an arbitrary threshold. Therefore, this method has the potential to identify multiple genes with small effects. If we assume that all individual tests are independent and arise from genetic variants with no effects, then *p*-values are identically and independently distributed as Uniform (0, 1). Taking this as a null hypothesis for the pattern of *p*-values in the global test, one can assess whether *p*-values, especially small *p*-values, are generated by chance. The global test of *p*-values is robust and can be applied to *p*-values from varying statistical models including *t*-tests, analysis of variance (ANOVA), linear mixed models, and so forth. Multiple simulation studies and case studies have demonstrated that this approach usually has sufficient power to detect signals of genetic association from a group of genes. For instance, Peng et al. (2010) has assessed Fisher's combination test and Sidak's combination test, Sime's combination test and the FDR method using 13 published genome wide association studies (GWAS), and the results indicate that combined *p*-value approaches can identify biologically meaningful pathways associated with the disease susceptibility. A review of methods of global test of *p*-values, developmental trends and their application to genetic data analysis has been presented by (Dai et al., 2012b).

One category of global tests of *p*-values involves combining *p*-values in the form of ∑*_i_H*(*p_i_*), where *p*-values might first be transformed by a function *H*. So far, several statistical methods have been developed to combine *p*-values. Let *p_i_*(*i* = 1,2,…, *n*) be independent *p*-values obtained from *n* hypothesis tests. Under the null hypothesis (*H*_0_) that *p*-values follow a Uniform (0, 1) distribution, Fisher (1932) shows that −2$\sum_{i=1}^{n}$ ln (*p_i_*) follows a chi-square distribution with 2*n* degrees of freedom. For a one sided test with a nominal error rate of α, one can reject the null hypothesis when the test statistics exceeds the (1 − α)^*^100% percentile of χ^2^_2*n*_. Stouffer (Stouffer et al., 1949) proposed a *z*-test by transforming *p*-values to standard normal variables, i.e., $\sum_{i=1}^{n}\frac{\Phi^{-1}(1-p_{i})}{\sqrt{n}}$, where Φ^−1^ is the inverse Cumulative Distribution Function (CDF) for *N*(0, 1). Under the null hypothesis, the *z*-test statistic follows *N*(0, 1).

Although there is no consensus regarding the most powerful method of combining *p*-values, Littell and Folks (1971, 1973) demonstrated that the Fisher's method of combining independent tests is asymptotically Bahadur efficient (Bahadur, 1967). Subsequently, weighting schemes have been incorporated into the Fisher's method and the *z*-test. Lancaster (1961) generalized the Fisher method by converting independent *p*-values to chi-square variables with *w_i_* degrees of freedom and he showed that $\sum_{i=1}^{m}\gamma_{\left(w_{i}/2,2\right)}^{-1}(1-p_{i})\sim \chi_{d}^{2}$, *d* = ∑*_i_w_i_* under *H*_0_, where γ^−1^_(*w_i_*/2,2)_ is the inverse CDF of Gamma distribution. Mosteller and Bush (1954) proposed a weighted *z*-test, $\sum_{i}w_{i}\Phi^{-1}(1-p_{i})/\sqrt{\sum_{i}w_{i}^{2}}$ which follows *N*(0,1) under *H*_0_.

In a separate paper, we have proved that the Lancaster procedure achieves the optimal Bahadur efficiency. We further demonstrated that the Lancaster procedure yields higher Bahadur efficiency than the weighted *z*-test. The Bahadur efficiency ratio gives the limiting ratio of sample sizes required by two statistics to attain an equally small significance level. Thus, Bahadur efficiency is an important method to compare test statistics. From the perspective of Bahadur efficiency, the Lancaster procedure asymptotically requires a relatively smaller sample size than other weighted *p*-value combining methods. This prompted us to focus on modification of the Lancaster procedure for correlated genetic data in this work.

Although the Fisher's method and Lancaster procedure both achieve the optimal Bahadur efficiency, the Lancaster procedure is more general and can be viewed as a generalized Fisher's method with weighting functions. There are three advantages to carefully select appropriate weight functions in genetic data analysis. Firstly, weight functions allow incorporation of prior biological information. Genetic data are complex and can be measured from different sources. Thus, weight functions can be used as a tool to incorporate meaningful information from different sources in order to interpret and derive biological insight from gene expression profiles. (Wu and Lin, 2009) provides a review of statistical methods for analysis of microarray data by incorporating prior biological knowledge using gene sets and biological pathways, which consist of groups of biologically similar genes. They show that the use of prior knowledge has led to a better understanding of the biological mechanisms underlying phenotypic responses. Secondly, weight functions can be used to remove bias. For instance, larger genes may contain more probes and/or SNPs. Therefore, larger genes will exert a stronger influence on the *p*-value combining methods as compared to smaller genes (Wang et al., 2007). To avoid this bias, one can consider a weight function to adjust for gene size when combining *p*-values. We will illustrate this approach in sections Empirical Assessments and Case Study: Renal Transplant Tolerance Data. Thirdly, as suggested by Benjamini and Hochberg (1997), Genovese et al. (2006), procedures that assign weights positively associated with the underlying alternative hypotheses will usually improve power. Therefore, one needs to carefully choose an appropriate weight function, either based on the biological knowledge, or by statistical hypotheses. An arbitrary weight is inappropriate for the Lancaster procedure.

In this work, we will provide modifications to the Lancaster procedure to accommodate correlation structures among *p*-values. The proposed method provides a generalization to the Fisher's method with a weight function and can be used in pathway analysis and gene sets enrichment analysis for a variety of genetic data including microarray gene expression data, GWAS data, and next generation sequencing data. In essence, investigators first dissect genetic variants by biological functions or prior knowledge, then combine the *p*-values from these gene sets to identify whether a proportion of genetic variants are associated with traits.

## Correlated lancaster procedures

In this section, we allow *p*-values to be correlated. Consider a Lancaster test statistic $T=\sum_{i=1}^{n}\gamma_{\left(w_{i}/2,2\right)}^{-1}(1-p_{i})$ where γ^−1^_(*w_i_* /2,2)_ is the inverse CDF of Gamma distribution with a shape parameter *w_i_ /2* and a scale parameter 2. This transformation converts *p_i_* ~ Uniform(0,1) to a chi-square distribution, i.e., γ^−1^_(*w_i_* /2, 2)_ (1−*p_i_*)~ χ^2^*_w_i__* where χ^2^*_w_i__* is a chi-square distribution with *w_i_* >0 degree(s) of freedom. The parameter *w_i_* serves as a weight function to adjust the individual *p*-values. When *p*-values are independent, *T* has an exact chi-square distribution with $\sum_{i=1}^{n}w_{i}$ degrees of freedom.

For correlated *p*-values, $T=\sum_{i=1}^{n}\gamma_{\left(w_{i}/2,2\right)}^{-1}(1-p_{i})$ does not follow $\chi_{\sum_{i=1}^{n}w_{i}}^{2}$. The distribution of *T* does not have an explicit analytical form. To address this issue, we consider a Satterthwaite approximation by approximating a scaled *T* statistic with a new chi-square distribution (Li et al., 2011). Let *cT* ≈ χ^2^*_v_* where *c* > 0 is a scalar and *v* > 0 is the degree of freedom for the approximated chi-square distribution. Note that

$$\begin{array}{l} \;E(T)=E\text{​}\left(\sum_{i=1}^{n}\gamma_{\left(w_{i}/2,2\right)}^{-1}\left(1-p_{i}\right)\right)=\sum_{i=1}^{n}w_{i}\text{ and}\\ \text{Var}(T)=\text{var​}\left(\sum_{i=1}^{n}\gamma_{\left(w_{i}/2,2\right)}^{-1}\left(1-p_{i}\right)\right)\\ \;=\sum_{i=1}^{n}\text{var}\text{​}\left(\gamma_{\left(w_{i}/2,2\right)}^{-1}\left(1-p_{i}\right)\right)\\ \;+2\sum_{i<j}\text{cov}\text{​}\left(\gamma_{\left(w_{i}/2,2\right)}^{-1}\left(1-p_{i}\right),\gamma_{\left(w_{i}/2,2\right)}^{-1}\left(1-p_{j}\right)\right)\\ \;=2\sum_{i=1}^{n}w_{i}+2\sum_{i<j}\rho_{ij}, \end{array}$$

where $\rho_{ij}=\text{cov}\left(\gamma_{\left(w_{i}/2,2\right)}^{-1}(1-p_{i}),\gamma_{\left(w_{i}/2,2\right)}^{-1}\left(1-p_{j}\right)\right)$ takes the correlations among *p*-values into account.

We propose the following five approaches to approximate the distribution of *T*. In approximation (A), we use the Satterthwaite method to match the mean and variance of *cT* and χ^2^*_v_*, and then solve the equations to derive *c* and *v*. Koziol (1996) have proposed multiple methods to approximate the Lancaster procedure, but these approximations require the assumption of independence. In approximation (B)–(E), we extend the work of Koziol (1996) to correlated data by first approximating *cT* with χ^2^*_v_* then approximating χ^2^*_v_* using varying methods.

- *T_A_* approximation.
  Correlation among *p*-values is taken into consideration, and then Satterthwaite's approximation is used (Patnaik, 1949) to derive new degrees of freedom:
  $$T_{A}=cT\approx \chi_{v}^{2},\text{ where }c=\frac{v}{E(T)}\text{ and }v=2\frac{{\left[E(T)\right]}^{2}}{\text{var}(T)}.$$
- *T_B_* approximation.
  *cT* is first approximated by χ^2^_*v*_, followed by Fisher's approximation (Fisher, 1922) to χ^2^_*v*_:
  $$T_{B}=\sqrt{2\frac{vT}{E(T)}}\approx N\text{​}(\sqrt{2v-1},1).$$
- *T_c_* approximation.
  After approximating *cT* by χ^2^_*v*_, the Wilson–Hilferty approximation is performed (Wilson and Hilferty, 1931) to derive χ^2^_*v*_.
  $$\text{Let }T_{c}=\sqrt[{3}]{\frac{T}{E(T)}},\text{ then }T_{c}\approx N\text{​}\left(1-2/(9v),\sqrt{2/(9v)}\right).$$
- *T_D_* approximation.
  Approximate *cT* by χ^2^_*v*_, followed by the Cornish–Fisher expansion (Fisher and Cornish, 1960) to χ^2^_*v*_. Let *x*_α_ denote the α-percentage point of the standard normal distribution, that is, Φ(*x*_α_) = α. It follows that the corresponding percentage point for $T_{D}=\frac{vT}{E(T)}$ is given by
  $$\begin{array}{l} v+\sqrt{2v}x_{\alpha }+\frac{2}{3}(x_{\alpha }^{2}-1)+\frac{x_{\alpha }^{3}-7x_{\alpha }}{9\sqrt{2v}}-\frac{6x_{\alpha }^{4}+14x_{\alpha }^{2}-32}{405v}\\ \;+\frac{9x_{\alpha }^{5}+256x_{\alpha }^{2}-433x_{\alpha }}{4860v\sqrt{2v}}. \end{array}$$
- *T_E_* approximation.
  Approximate *cT* by χ^2^_*v*_ then perform saddle point approximation (Lugannani and Rice, 1980) to χ^2^_*v*_. Let $T_{E}=\frac{T}{E(T)}$. Then Pr (*Y_E_* ≤ *y*) = Φ (*a_y_*) − ϕ(*b_y_*^−1^ − *a_y_*^−1^) for *y* ≠ 1 and $\mathrm{Pr}(Y_{E}\leq 1)=0.5-{\left(3\sqrt{\pi v}\right)}^{-1}$, where $a_{y}=\sqrt{2v(yt_{y}-K(t_{y}))\text{sign}(t_{y})}$, $b_{y}=t_{y}\;\sqrt{vK"(t_{x})}$ and *K(t)* = −0.5log (1 − 2t), and *t_y_* = (*y* − 1)/2*y*.

When the covariance ρ*_ij_* is unknown, one can use the permutation approach to estimate ρ*_ij_* by shuffling the phenotype variable among subjects. For the *k*th permutation (*k* = 1,2,…,*m*), we keep the genetic variants within the subject to preserve the correlation structure, then randomly assign the phenotype variable to subjects. Individual hypothesis testing can be done on all *n* genetic variants separately to generate the *p*-value vector *p^k^* = (*p^k^*_1_, *p^k^*_2_,… *p^k^_n_*)*^t^*. The permutation is repeated *m* = 1000 times, and ρ*_ij_* is estimated from (*p*^1^, *p*^2^, … *p^m^*).

The accuracy of the five approximate distributions to the correlated Lancaster procedure is then assessed using *p*-values with varying correlation structures. We consider six different types of correlation structures, including fixed and random compound symmetric as well as random positive definite variance-covariance structures for Σ. Let *I* be an identity matrix, $\vec{1}$ be a vector of 1 s, ⊗ be the Kronecker product, and superscript *t* be the transposition. In Cases I–V, let Σ = Block ⊗ *I*_20_ be compound symmetric variance matrices with 20 blocks of size 5 where $\text{Block}={\vec{1}}_{5}{\vec{1}}_{5}^{t}\rho +(1-\rho )I_{5}$. We vary ρ over two fixed values with ρ = 0.3 for moderate dependence and ρ = 0.6 for strong dependence. In addition, we simulate random correlation coefficients from beta and uniform distributions, i.e., ρ ~ β(0.3, 1.5) and ρ ~ uniform(−0.2, 0.2), which ensures that 20 variance blocks have distinct correlation coefficients ρ within Σ. More generally, we consider random positive definite correlation matrices Σ that vary across samples and simulation runs.

**Figure 1:**
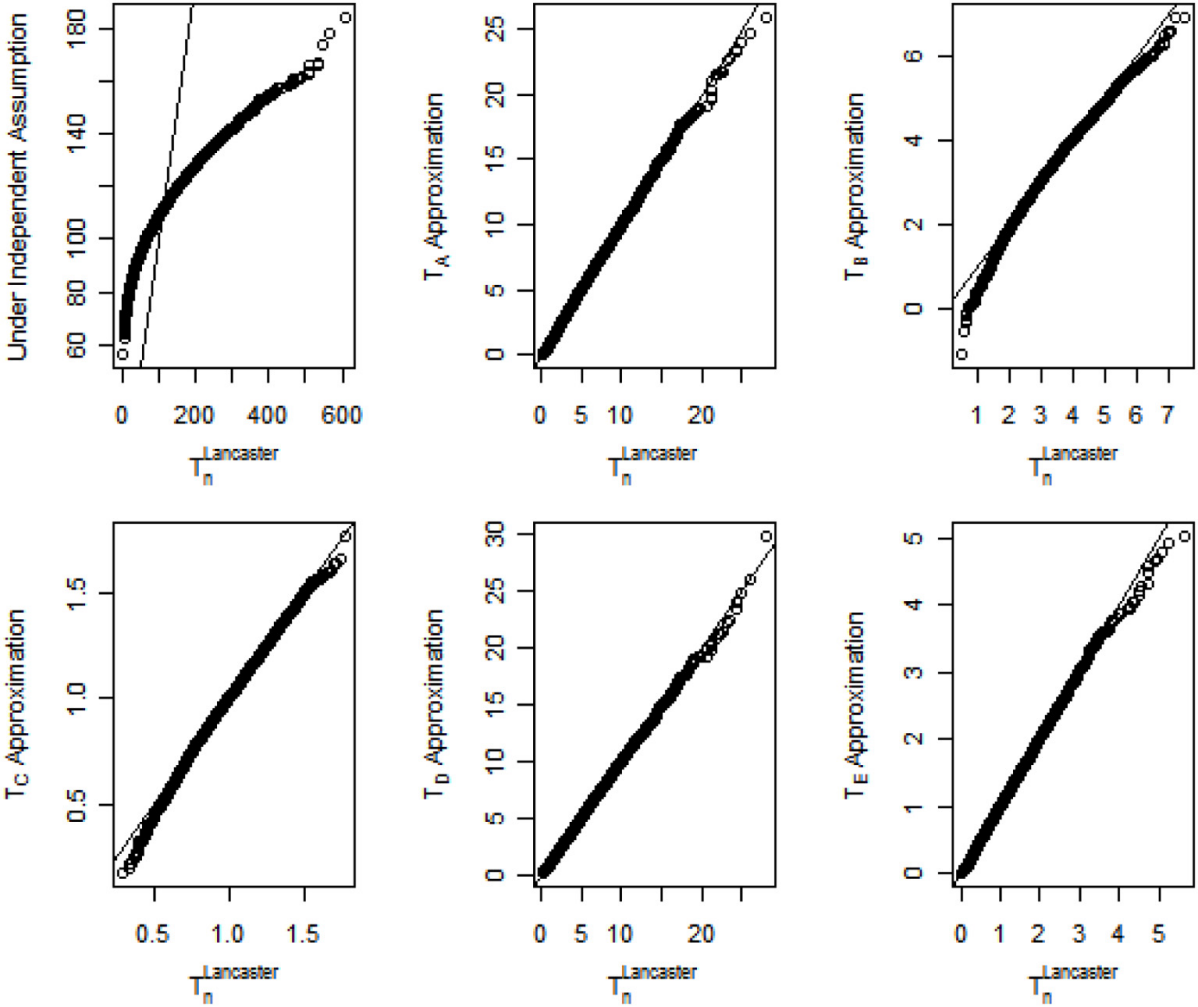
**Q-Q plots for distributions of the Lancaster statistic when *p*-values are correlated with correlation coefficient ρ = 0.3**.

The quantile-quantile (Q-Q) plot assessing the accuracy of the proposed methods when the correlation coefficient ρ = 0.3 is shown in Figure 1. For clarity, the Lancaster statistic *T* that combines *n p*-values is renamed as *T^Lancaster^_n_* in Figure 1. For the original Lancaster procedure under the independence assumption, the general trend of the Q-Q plot is flatter than the reference line *y* = *x*, indicating the limiting distribution for the test statistic in the original Lancaster procedure is less dispersed than the distribution of *T^Lancaster^_n_* under correlation structures. As a result, the original Lancaster procedure will have severely inflated Type I errors. In contrast, the five approximations (*T_A_*, …, *T_E_*) match the underlying distribution of *T^Lancaster^_n_*. For data with stronger internal correlation, *T_A_*, *T_D_*, and *T_E_* better approximate *T^Lancaster^_n_*. The Q-Q plots under other correlation structures are similar to Figure 1. To save space, these similar results are not shown, but can be provided upon request.

## Empirical assessments

We assess the Type I error rates and power for the proposed correlated Lancaster procedures and compare them to the independent Lancaster procedure (Lancaster, 1961). SNPs from a pathway of haploid GWAS are simulated using linkage disequilibrium (LD) (Li et al., 2011). Let *q*_1_ and *q*_2_ be the minor allele frequencies (MAFs) at loci 1 and 2. Assuming Hardy–Weinberg equilibrium, the genotype at locus 1 can be randomly generated using a binomial distribution. Given the distribution of SNP at locus 1, one can simulate the genotype at locus 2. To do so, let *D* be a measure of LD. Then the conditional probability for the genotype at locus 2 given the genotype at locus 1 can be expressed as *P*(*A*|*B*) = [*q_A_ q_B_* + *D*]/*q_B_*, *P*(*a*|*B*) = [(1−*q_A_*)*q_B_* −*D*]/*q_B_*, *P*(*A*|*b*) = [*q_A_* (1−*q_B_*)−*D*]/(1−*q_B_*), and *P*(*a*|*b*) = [(1−*q_A_*)(1−*q_B_*)+*D*]/(1−*q_B_*) where *A* and *B* represent the minor alleles at the two loci. For a diploid genome, similar idea can be applied and the simulation details can be found at Cui et al. (2008). We simulate a pathway with 5 genes with varying numbers of SNPs in each gene listed in parenthesis i.e., G1(12), G2(8), G3(5), G4(3), G5(2). The MAF of each SNP was set to be 0.3. We simulate different levels of LD for SNPs from the same gene with *D* = 0, 1.5, 2, and uniform(0, maximum of LD). The variable *D* = 0, 1.5, and 2 suggests no LD, moderate LD, and very strong LD among SNPs with the corresponding correlation *R* = 0, 0.71, and 0.95. Six scenarios for disease susceptibility (*p*) are simulated

- Case I: ln (*p*/(1−*p*)) = β_1_ *G*_1, 2_ +β_2_ *G*_1, 5_ +β_3_ *G*_1, 7_ +β_4_ *G*_1, 8_ +β_5_ *G*_1, 12_.
- Case II: ln (*p*/(1−*p*)) = β_1_ *G*_2, 2_ +β_2_ *G*_2, 4_ +β_3_ *G*_2, 6_ +β_4_ *G*_3, 2_ +β_5_ *G*_3, 3_.
- Case III: ln (*p*/(1−*p*)) = β_1_ *G*_3, 2_ +β_2_ *G*_3, 4_ +β_3_ *G*_4, 1_ +β_4_ *G*_4, 3_ +β_5_ *G*_5, 1_.
- Case IV: ln (*p*/(1−*p*)) = β_1_ *G*_1, 1_ +β_2_ *G*_1, 3_ +β_3_ *G*_1, 7_ +β_4_ *G*_1, 8_ *G*_1, 10_ *G*_1, 11_ +β_5_ *G*_1, 12_.
- Case V: ln (*p*/(1−*p*)) = β_1_ *G*_3, 1_ +β_2_ *G*_3, 3_ +β_3_ *G*_4, 2_ +β_4_ *G*_3, 2_ *G*_3,4_ +β_5_ *G*_4, 3_ *G*_5, 1_.
- Case VI: ln (*p*/(1−*p*)) = β_1_ *G*_1, 2_ +β_2_ *G*_2, 2_ +β_3_ *G*_3, 3_ +β_4_ *G*_5, 2_ +β_5_ *G*_1, 5_ *G*_1,7_ +β_6_ *G*_3, 3_ *G*_5, 1_.

Weight functions can be used to remove potential bias when combining *p*-values. Wang et al. (2007) and others have noted that larger genes contain more probes and/or SNPs. Therefore, larger genes may exert a stronger influence on the *p*-value combining methods compared to smaller genes. To avoid this bias, we set the weight function $w_{i}=2/\sqrt{n_{i}}$ where *n_i_* is the number of SNPs in the *i*th gene. When *n_i_* = 1, γ^−1^_(*w_i_* /2, 2)_ (1−p_i_) transforms *p*-value into a variable with χ^2^_2_ distribution.

We simulate data with sample sizes *n* = 200 (Tables 1, 4) and *n* = 400 (Tables 2, 3), respectively. For simplicity, we assume the same effect size for all of the regression coefficients. For each set of data, we perform the original and modified Lancaster procedures to assess the pathway data by combining *p*-values from individual tests. We set nominal error rate to be 0.05. The simulation is repeated 1000 times.

**Table 1:** **Type I error and power for independent Lancaster Procedure and five approximations to correlated Lancaster Procedures when sample size = 200 and linkage disequilibrium *D* = 0.15**.

	**Independent Lancaster procedure**	***T_A_***	***T_B_***	***T_C_***	***T_D_***	***T_E_***
**CASE I**						
β = 0	*0.101*	0.038	0.042	0.039	0.039	0.038
β = 0.4	0.999	0.995	0.995	0.995	0.995	0.995
β = 0.6	1	1.000	1	1	1	1
**CASE II**						
β = 0	*0.1*	0.037	0.041	0.038	0.038	0.037
β = 0.4	0.947	0.863	0.875	0.864	0.865	0.863
β = 0.6	0.997	0.995	0.995	0.995	0.995	0.995
**CASE III**						
β = 0	*0.078*	0.038	0.038	0.038	0.038	0.038
β = 0.4	0.735	0.506	0.522	0.508	0.507	0.506
β = 0.6	0.961	0.864	0.876	0.866	0.866	0.863
**CASE IV**						
β = 0	*0.107*	0.046	0.051	0.046	0.047	0.046
β = 0.4	0.997	0.997	0.997	0.997	0.997	0.997
β = 0.6	1	1	1	1	1	1
**CASE V**						
β = 0	*0.084*	0.036	0.038	0.037	0.037	0.036
β = 0.4	0.884	0.71	0.724	0.71	0.711	0.71
β = 0.6	0.989	0.952	0.957	0.953	0.953	0.952
**CASE VI**						
β = 0	*0.084*	0.036	0.038	0.037	0.037	0.036
β = 0.4	0.741	0.57	0.585	0.572	0.572	0.568
β = 0.6	0.953	0.898	0.904	0.898	0.898	0.898

A weight function is applied to adjust for the gene size^*^.

^*^The nominal error rate is set to be 0.05. Type I error rates are listed when β = 0. Power is listed when β > 0. Inflated Type I error rates are italicized.

^*^A weight function $w_{i}=2/\sqrt{n_{i}}$ is applied to each test to adjust for the size of gene.

**Table 2:** **Type I error and power for independent Lancaster Procedure and five approximations to correlated Lancaster Procedures when sample size = 400 and linkage disequilibrium *D* = 0.20**.

	**Independent Lancaster procedure**	***T_A_***	***T_B_***	***T_C_***	***T_D_***	***T_E_***
**CASE I**						
β = 0	*0.13*	0.051	0.052	0.051	0.051	0.051
β = 0.4	1	1	1	1	1	1
β = 0.6	1	1	1	1	1	1
**CASE II**						
β = 0	*0.134*	0.05	0.051	0.05	0.05	0.05
β = 0.4	0.999	0.997	0.998	0.998	0.998	0.997
β = 0.6	1	1	1	1	1	1
**CASE III**						
β = 0	*0.116*	0.045	0.048	0.045	0.045	0.045
β = 0.4	0.986	0.908	0.915	0.908	0.908	0.908
β = 0.6	1	0.998	0.998	0.998	0.998	0.998
**CASE IV**						
β = 0	*0.109*	0.046	0.047	0.046	0.046	0.046
β = 0.4	1	1	1	1	1	1
β = 0.6	1	1	1	1	1	1
**CASE V**						
β = 0	*0.135*	0.04	0.043	0.041	0.041	0.041
β = 0.4	0.994	0.971	0.974	0.971	0.971	0.971
β = 0.6	1	1	1	1	1	1
**CASE VI**						
β = 0	*0.135*	0.04	0.043	0.041	0.041	0.041
β = 0.4	0.986	0.939	0.948	0.939	0.939	0.939
β = 0.6	1	0.999	0.999	0.999	0.999	0.999

A Weight function is applied to adjust for the gene size^*^.

^*^The nominal error rate is set to be 0.05. Type I error rates are listed when β = 0. Power is listed when β > 0. Inflated Type I error rates are italicized.

^*^A weight function $w_{i}=2/\sqrt{n_{i}}$ is applied to each test to adjust for the size of gene.

**Table 3:** **Type I error and power for independent Lancaster Procedure and five approximations to correlated Lancaster Procedures when sample size = 400 and linkage disequilibrium *D* = 0.15**.

	**Independent Lancaster procedure**	***T_A_***	***T_B_***	***T_C_***	***T_D_***	***T_E_***
**CASE I**						
β = 0	*0.066*	0.043	0.045	0.043	0.044	0.043
β = 0.4	0.991	0.978	0.978	0.978	0.978	0.978
β = 0.6	1	1	1	1	1	1
**CASE II**						
β = 0	*0.059*	0.031	0.035	0.031	0.031	0.031
β = 0.4	0.978	0.964	0.967	0.964	0.964	0.964
β = 0.6	1	1	1	1	1	1
**CASE III**						
β = 0	*0.053*	0.029	0.034	0.029	0.03	0.029
β = 0.4	0.898	0.836	0.844	0.837	0.837	0.836
β = 0.6	0.999	0.996	0.997	0.996	0.996	0.996
**CASE IV**						
β = 0	*0.072*	0.041	0.045	0.041	0.041	0.041
β = 0.4	0.977	0.962	0.964	0.962	0.962	0.962
β = 0.6	1	1	1	1	1	1
**CASE V**						
β = 0	*0.072*	0.041	0.045	0.041	0.041	0.041
β = 0.4	0.946	0.899	0.905	0.9	0.901	0.899
β = 0.6	0.999	0.996	0.996	0.996	0.996	0.996
**CASE VI**						
β = 0	*0.072*	0.041	0.045	0.041	0.041	0.041
β = 0.4	0.807	0.732	0.045	0.733	0.733	0.732
β = 0.6	0.978	0.965	0.045	0.965	0.965	0.965

A weight function is applied to adjust for the gene size^*^.

^*^The nominal error rate is set to be 0.05. Type I error rates are listed when β = 0. Power is listed when β > 0. Inflated Type I error rates are italicized.

^*^A weight function $w_{i}=2/\sqrt{n_{i}}$ is applied to each test to adjust for the size of gene.

Due to LD, SNPs from the same gene are correlated. We first assess the Type I error rate of the test statistics by testing *H*_0_:β_1_ = … = β_6_ = 0. As shown in Tables 1, 2, the Type I error rate for the original Lancaster procedure is inflated (>0.05) for all of the six cases. In contrast, five modified Lancaster procedures (*T_A_* − *T_E_*) have well controlled Type I error rates (<0.05).

The power of all test statistics was compared for regression coefficient values set at β = 0.4 and β = 0.6, respectively. The results in Tables 1, 2 suggest strong and comparable power among the modified Lancaster procedures. In most simulated cases, the proposed methods have more than 80% power to detect β = 0.4. When the effect size increases to β = 0.6, the power of proposed methods increases to 90% or above. Also the power of these tests improves as sample size increases from *n* = 200 to *n* = 400.

We simulate different levels of LD for SNPs with *D* = 0, 1.5, 2, and uniform(0, maximum of LD). To save the space, we only show the results for *D* = 1.5 (Table 3) and *D* = 2 (Tables 1, 2). Our findings show that the inflation of Type I error rate for the original Lancaster procedure gets severe when LD is strong (Tables 1, 2). The modified Lancaster procedures (*T_A_* − *T_E_*) have well-controlled Type I error rates and power for both moderate and strong LD (Tables 1–3).

In Table 4, we assess the performance of all tests without a weighting function. We then compare the results in Table 4 (without a weight function) vs. Table 1 (with a weight function). All other simulation parameters are held the same in Tables 1, 4. We note that the original Lancaster procedure without a weighting function (Table 4) tends to have higher Type I error rates than the original Lancaster procedure with a weighting function (Table 1). For modified tests (*T_A_* − *T_E_*), the power is increased when a weighting function is used. This confirms that an appropriate weight function is beneficial to the Lancaster procedure.

**Table 4:** **Type I error and power for independent Lancaster Procedure and five approximations to correlated Lancaster Procedures when sample size = 200 and linkage disequilibrium *D* = 0.20**.

	**Independent Lancaster procedure**	***T_A_***	***T_B_***	***T_C_***	***T_D_***	***T_E_***
**CASE I**						
β = 0	*0.106*	0.027	0.03	0.027	0.027	0.027
β = 0.4	1	0.997	0.997	0.997	0.997	0.997
β = 0.6	1	1	1	1	1	1
**CASE II**						
β = 0	*0.1*	0.029	0.03	0.029	0.029	0.029
β = 0.4	0.935	0.801	0.812	0.801	0.803	0.801
β = 0.6	0.998	0.976	0.98	0.976	0.977	0.976
**CASE III**						
β = 0	*0.118*	0.041	0.042	0.041	0.041	0.041
β = 0.4	0.608	0.307	0.32	0.307	0.307	0.307
β = 0.6	0.881	0.663	0.679	0.665	0.666	0.663
**CASE IV**						
β = 0	*0.115*	0.037	0.04	0.038	0.038	0.037
β = 0.4	1	0.994	0.994	0.994	0.994	0.994
β = 0.6	1	1	1	1	1	1
**CASE V**						
β = 0	*0.115*	0.037	0.04	0.038	0.038	0.037
β = 0.4	0.78	0.487	0.5	0.488	0.489	0.487
β = 0.6	0.977	0.869	0.882	0.869	0.87	0.869
**CASE VI**						
β = 0	*0.115*	0.037	0.04	0.038	0.038	0.037
β = 0.4	0.782	0.579	0.589	0.579	0.58	0.579
β = 0.6	0.964	0.885	0.888	0.885	0.885	0.885

No Weight function is applied to adjust for the gene size^*^.

^*^The nominal error rate is set to be 0.05. Type I error rates are listed when β = 0. Power is listed when β > 0. Inflated Type I error rates are italicized.

^*^These are the un-weighted tests with *w_i_* = 2 for all genes. We do not adjust the size of genes.

## Case study: renal transplant tolerance data

We revisited a kidney transplant data first collected and analyzed by Newell et al. (2010). Data were downloaded from the GEO website with ID = GDS4266 (http://www.ncbi.nlm.nih.gov/sites/GDSbrowser?acc=GDS4266). A group of tolerant renal transplant recipients (Tolerant, *n* = 19), as defined by stable graft function in the absence of immunosuppression for more than 1 year, were compared to subjects with stable graft function who were receiving standard immunotherapy (SI, *n* = 27) as well as to a group of healthy controls (Control, *n* = 12). Gene expression profiles of whole-blood total RNA from all subjects were measured by microarray. The goal of the study was to identify genetic variants associated with long-term allograft survival without the requirement for continuous immunosuppression, a condition known as allograft tolerance. Newell et al. (2010) performed statistical analysis to identify differentially expressed genes between the SI group and the Tolerant group. The results revealed a critical role for B cells in regulating alloimmunity, and provided a candidate set of genes for wider-scale screening of renal transplant recipients. However, no comprehensive pathway analysis was conducted by this group (Newell et al., 2010).

To further understand molecular mechanisms underlying renal allograft tolerance, we have applied the modified Lancaster procedure to this dataset to identify candidate cellular pathways. Gene expression levels were normalized using Robust Multichip Average (rma) preprocessing methodology, which included background subtraction, quantile normalization, and summarization via median-polish.

Gene expression levels were summarized for a total of 54,675 probes from 21,049 genes. Expression levels were compared among three groups using the Bioconductor “Limma” package. Three pair wise comparisons were conducted, including: SI vs. Control, SI vs. Tolerant, and Tolerant vs. Control. Then three comparisons were combined into one *F*-test. This is equivalent to a One-Way ANOVA for each gene except that the residual mean squares have been moderated across genes. *P*-values from multiple hypothesis testing were adjusted by FDR (Benjamini and Hochberg, 1995). Our results of differentially expressed genes are consistent with the previous published work. See Newell et al. (2010) for the gene analysis findings.

Although (Newell et al., 2010) identified a set of differentially expressed genes, our analysis demonstrates that these significant genes have small effect sizes with fold changes <1.5. Therefore, a limited number of individual genes in the absence of a biological context is inadequate to explain the total variation of allograft tolerance among renal transplant patients.

To address this issue, we performed the modified Lancaster procedure (*T_A_*) as described in Section Correlated Lancaster Procedures to combine *p*-values from pathways. Combining *p*-values allows us to integrate small effects in pathway and gain the power of statistical testing. A total of 1454 Gene Ontology human pathway gene sets were analyzed. The size of pathways ranged from 9 genes to 2131 genes, with a median of 27 genes per pathway. Also, the number of probes per gene was highly variable. In order to map genes to pathways, we removed genes without gene symbols from the analysis. Among 21,049 genes with gene symbols, approximately 48% (*n* = 10161) of genes were interrogated with a single probe, 26% (*n* = 5389) of genes were queried using 2 probes, 14% (*n* = 2842) of genes were assessed by 3 probes. There were 3 or more probes for each on the remaining genes (range: 4–17). This finding indicates that larger genes would have more *p*-values and a stronger impact to pathway analysis. To prevent this bias, we set the weight function as $w_{i}=2/\sqrt{n_{i}}$ where *n_i_* is the number of probes for the *i*th gene.

We performed pathway analysis for the One-Way ANOVA test and three pair wise comparisons. The top 10 significant pathways based on the One-Way ANOVA test are listed in Table 5. The top two pathways, B cell differentiation (GO:0030183) and B cell activation (GO:0042113), confirm the signature of B cell involvement described by Newell et al. (2010). Furthermore, we identified other pathways related to B cell activation and function. These include antigen binding (GO:0003823), map kinase kinase kinase activity (GO:0004709) and lymphocyte differentiation (GO:0030098). These pathways are biologically consistent with the proposed role of B-lymphocytes in renal transplant tolerance reported by Newell et al. In contrast, when we performed the traditional Fisher's method without considering correlation structures (LD) within pathways or applying a weighting function to compensate for variability in the number of probes per gene, the result was a list of larger pathways, some containing >1000 genes, describing more general cellular processes and not specifically related to immune functions (See Table 6, #gene and #probe). Furthermore, when comparing the SI group and the Control group, the traditional method identified 1078 significant pathways while our proposed method narrowed the list down to 64 significant pathways (adjusted *p*-value <0.05). The increase in number of significant pathways identified by the traditional approach is primarily due to false positive discovery, and is consistent with the inflation of Type I error rate as presented in Section Empirical Assessments. Thus, by accounting for correlation structures (LD) within pathways and the number of probes per gene, our proposed method minimized identification of larger, non-specific cellular processes pathways, and instead revealed more focused and functionally relevant biological pathways implicating a role for a humoral immune response in immunotolerance to renal transplants (See Table 5, #gene and #probe).

**Table 5:** **Top 10 significant pathways detected by the modified Lancaster procedure (*T_A_*)**.

**GO accession**	**Pathway name**	**Gene ontology**	**URL**	#**Gene**	#**Probe**	**Adjusted *P*-value**
GO:0030183	B cell differentiation	Biological process	http://www.broadinstitute.org/gsea/msigdb/cards/B_CELL_DIFFERENTIATION	12	29	0.003541
GO:0042113	B cell activation	Biological process	http://www.broadinstitute.org/gsea/msigdb/cards/B_CELL_ACTIVATION	20	45	0.003541
GO:0003823	Antigen binding	Molecular function	http://www.broadinstitute.org/gsea/msigdb/cards/ANTIGEN_BINDING	23	51	0.003541
GO:0004709	Map kinase kinase kinase activity	Molecular function	http://www.broadinstitute.org/gsea/msigdb/cards/MAP_KINASE_KINASE_KINASE_ACTIVITY	10	32	0.003541
GO:0017148	Negative regulation of translation	Biological process	http://www.broadinstitute.org/gsea/msigdb/cards/NEGATIVE_REGULATION_OF_TRANSLATION	23	36	0.003541
GO:0042493	Response to drug	Biological process	http://www.broadinstitute.org/gsea/msigdb/cards/RESPONSE_TO_DRUG	20	35	0.004669
GO:0001772	Immunological synapse	Cellular component	http://www.broadinstitute.org/gsea/msigdb/cards/IMMUNOLOGICAL_SYNAPSE	10	18	0.006603
GO:0030098	Lymphocyte differentiation	Biological process	http://www.broadinstitute.org/gsea/msigdb/cards/LYMPHOCYTE_DIFFERENTIATION	26	53	0.007986
GO:0042036	Negative regulation of cytokine biosynthetic process	Biological process	http://www.broadinstitute.org/gsea/msigdb/cards/NEGATIVE_REGULATION_OF_CYTOKINE_BIOSYNTHETIC_PROCESS	12	21	0.008582
GO:0009890	Negative regulation of biosynthetic process	Biological process	http://www.broadinstitute.org/gsea/msigdb/cards/NEGATIVE_REGULATION_OF_BIOSYNTHETIC_PROCESS	30	48	0.008582

**Table 6:** **Top 10 significant pathways detected by the traditional Fisher's method**.

**GO accession**	**Pathway name**	**Gene ontology**	**URL**	**# Gene**	**# Probes**	**Adjusted *P*-value**
						***P*-value**
GO:0005737	Cytoplasm	Cellular component	http://www.broadinstitute.org/gsea/msigdb/cards/CYTOPLASM	2078	4986	0.E+00
GO:0005634	Nucleus	Cellular component	http://www.broadinstitute.org/gsea/msigdb/cards/NUCLEUS	1393	3588	0.E+00
GO:0043283	Biopolymer metabolic process	Biological process	http://www.broadinstitute.org/gsea/msigdb/cards/BIOPOLYMER_METABOLIC_PROCESS	1653	4240	0.E+00
GO:0016020	Membrane	Cellular component	http://www.broadinstitute.org/gsea/msigdb/cards/MEMBRANE	1954	4395	3.E−307
GO:0006139	Nucleobase, nucleoside, nucleotide, and nucleic acid metabolic process	Biological process	http://www.broadinstitute.org/gsea/msigdb/cards/NUCLEOBASENUCLEOSIDENUCLEOTIDE_AND_NUCLEIC_ACID_METABOLIC_PROCESS	1217	3112	6.E−305
GO:0007165	Signal transduction	Biological process	http://www.broadinstitute.org/gsea/msigdb/cards/SIGNAL_TRANSDUCTION	1604	3826	1.E−296
GO:0044425	Membrane part	Cellular component	http://www.broadinstitute.org/gsea/msigdb/cards/MEMBRANE_PART	1638	3670	4.E−251
GO:0019538	Protein metabolic process	Biological process	http://www.broadinstitute.org/gsea/msigdb/cards/PROTEIN_METABOLIC_PROCESS	1205	3022	2.E−245
GO:0044422	Organelle part	Cellular component	http://www.broadinstitute.org/gsea/msigdb/cards/ORGANELLE_PART	1173	2934	1.E−230
GO:0044446	Intracellular organelle part	Cellular component	http://www.broadinstitute.org/gsea/msigdb/cards/INTRACELLULAR_ORGANELLE_PART	1168	2923	4.E–230

## Discussion and conclusions

Modifications to the Lancaster procedure are proposed to take correlations among *p*-values into account. Extensive simulation studies show that the original Lancaster procedure has inflated Type I error rates due to correlation among *p*-values. By using permutation approach to estimate the correlation among *p*-values, the proposed methods have well-controlled Type I error rates and maintain strong power to detect signals related to SNPs in pathways.

Among five proposed approximation methods (*T_A_*, …, *T_E_*), the Satterthwaite approximation (*T_A_*) is the most computationally efficient. Other approximation methods (*T_B_*, …, *T_E_*) are based on the Satterthwaite approximation. Therefore, we recommend using the Satterthwaite approximation (*T_A_*) as the standard procedure to modify the Lancaster procedure. Among other approximation methods, simulation results in Section Correlated Lancaster Procedures show that, for data with stronger internal correlation, *T_D_* and *T_E_* have better approximation than *T_B_* and *T_C_*. Our simulation study and the case study further provide evidence that *T_D_* tends to have slightly higher power than the Satterthwaite approximation *T_A_*. The R code for five approximation is posted at http://d.web.umkc.edu/daih/.

### Conflict of interest statement

The authors declare that the research was conducted in the absence of any commercial or financial relationships that could be construed as a potential conflict of interest.
